# Meeting of the McLean Co. Medical Society

**Published:** 1858-09

**Authors:** S. W. Noble, H. Noble

**Affiliations:** President; Secretary pro tem.


### PROCEEDINGS OF MEDICAL SOCIETIES.

### MEETING OF THE McLEAN CO. MEDICAL SOCIETY.

The society met in Le Roy on the 12th of July, 1858, and was called to order by the President, Dr. S. W. Noble.

The Secretary being absent, Dr. H. Noble acted as Secretary *pro tem.*

The constitution of the society was read by Dr. Luce and signed by Dr. Stewart and Dr. Laughlin, who had not before had an opportunity of signing the same.

Dr. S. W. Noble reported a case of cancerous disease of the stomach and liver, and exhibited the diseased structures.

Dr. Laughlin read an essay on the Improvement of Medical Science, which, on motion, was received and ordered to be put on file.

Dr. Fisher read an essay on the Ill Effects of the Use of Patent Medicines, which, after the usual reference, was discussed by Drs. Luce, S. W. Noble, Stewart, Fisher and Laughlin.

Dr. Stewart read an essay on Physiognomy, which was received and ordered to be put on file.

On motion of Dr. Luce, the thanks of the society were tendered to the Essayists for the prompt manner in which they discharged the duties imposed on them by the society.

The President appointed Drs. Coleman and Luce to read essays at the next meeting of the society.

By invitation of Dr. Laughlin, it was resolved that the society meet at Heyworth on the second Monday of October, 1858, at half-past ten o'clock a. m.

It was resolved that the Secretary be instructed to inform Drs. Worrell and Roe that they will have the privilege to continue and finish, at Heywood meeting, their discussion of Dr. Elder's paper, which was commenced at Bloomington on 12th April, and suspended by agreement to be continued at Le Roy meeting.

It was resolved that a committee of three be appointed to inquire into the matter of unprofessional conduct and violation of ethics, said to have been committed by Dr. Craig, a member of this society, and if they find sufficient reason therefor, that they prepare articles of impeachment against said Craig, and notify him that they will be presented to the society at its next quarterly meeting, to be held at Heyworth. Drs. Luce, Fisher and H. Noble were appointed said committee.

On motion of Dr. Stewart, it was resolved that the Secretary be instructed to transmit a copy of this meeting's proceedings to the *Chicago Medical Journal* for publication.

S. W. NOBLE, *President.*

H. NOBLE, *Secretary pro tem.*

